# 1267. Implementation and Outcomes of Beta-lactam Allergy Management Protocol at a Comprehensive Cancer Center

**DOI:** 10.1093/ofid/ofad500.1107

**Published:** 2023-11-27

**Authors:** Sohanabanu Khalak, Wonhee So, Stephanie Ho, Justine Abella Ross, Sanjeet S Dadwal, Alfredo Puing, Deepa D Nanayakkara, Randy Taplitz, Avneet Kaur, Jana Dickter

**Affiliations:** Western University of Health Sciences, Duarte, California; Western University of Health Sciences, Duarte, California; City of Hope National Medical Center, Duarte, California; City of Hope National Medical Center, Duarte, California; City of Hope National Medical Center, Duarte, California; COH, Duarte, California; COH, Duarte, California; City of Hope National Medical Center, Duarte, California; City of Hope Comprehensive Cancer Center, DUARTE, California; City of Hope National Medical Center, Duarte, California

## Abstract

**Background:**

Beta-lactam allergy (BLA) is associated with increased broad-spectrum antibiotic (Br-ABX) use, worse clinical outcomes, and higher costs. Our hospital-wide BLA protocol (BLA-P) was launched in 7/2021 with following categories: intolerance, low-risk (urticaria only >5 years ago, mild rash, remote childhood reaction with limited details), and high-risk (angioedema, anaphylaxis, severe rashes). Delabeling was done directly based on antibiotic history/interview alone (direct-delabeling), or via graded challenge for low-risk patients. We evaluated the delabeling rate and its impact on Br-ABX usage.

**Methods:**

Hospitalized patients ≥ 18 years old with listed BLA during 10/2021-12/2022 were eligible. Exclusion criteria were critically ill, surgical, hospice or comfort care, or non-verbal patients. Assessment was counted each time a pharmacist evaluated BLA; multiple assessments could occur in case of prolonged hospital stay or multiple admissions. Interventions were categorized as no further action (due to high-risk allergy, patient refusal for any update, clinical status), updated allergy label, or delabeled. Missed assessments were reported due to logistical issues. Br-ABX usage was compared in the delabeled patients: the empiric antibiotic use 90 days post-intervention versus pre-intervention using McNemar test (SPSS).

**Results:**

A total of 700 assessments in 631 unique patients with BLA were identified (Figure 1). 556 assessments in 489 unique patients (median age 63 years, 41% male, 46% hematological cancer) met inclusion criteria. In this cohort, the assessments revealed 8% intolerance, 54% low-risk, 17% high-risk and 21% unknown allergy. Interventions resulted in no further action 7%, updated label 72%, and delabeling 21%. 65% of the delabeling was done via direct-delabeling and 35% via graded challenge (Figure 1). Passing rate with graded challenge was 97%. The use of aztreonam and meropenem decreased significantly in delabeled patients compared to pre-delabeling while cefepime and piperacillin-tazobactam usage increased (Table 1).

**Figure:**
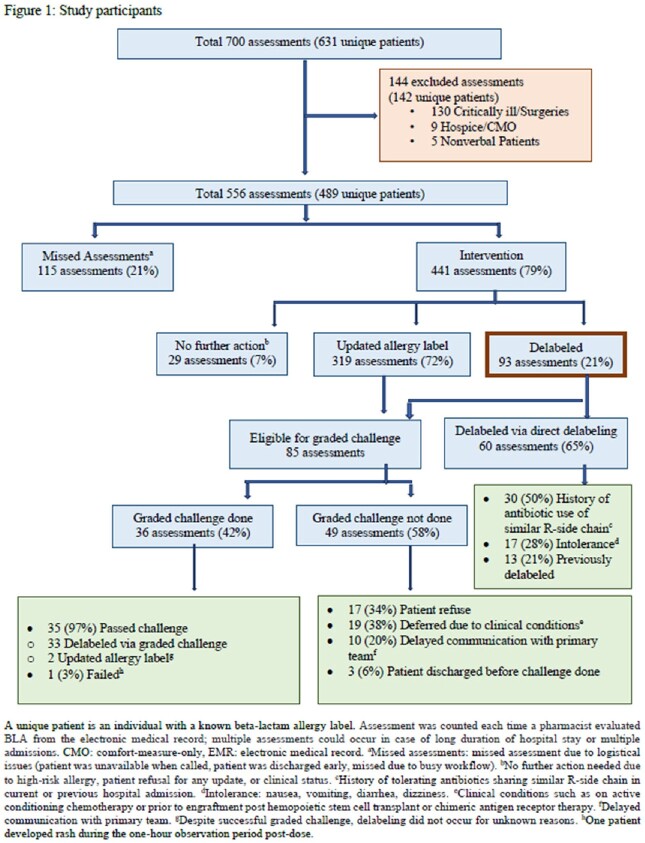

**Figure:**
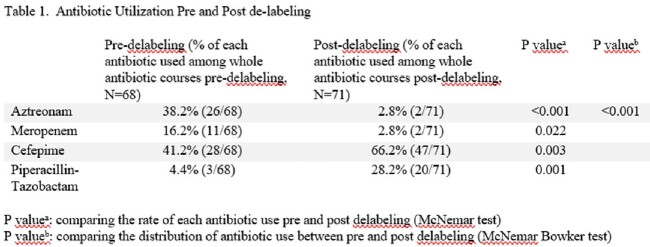

**Conclusion:**

Implementation of BLA-P led to 21% delabeling, which resulted in increased use of preferred Br-ABX and a significant decrease in meropenem and aztreonam use.

**Disclosures:**

**Sanjeet S. Dadwal, MD, FACP, FIDSA**, Allovir: Advisor/Consultant|Allovir: Grant/Research Support|Ansun Biopharma: Grant/Research Support|Aseptiscope, Inc: Stocks/Bonds|Astellas: Honoraria|Karius: Grant/Research Support|Matinas Biopharma: Stocks/Bonds|Merck: Advisor/Consultant|Merck: Grant/Research Support|Pfizer/Amplyx: Grant/Research Support|Takeda: Advisor/Consultant|Takeda: Honoraria|Viracor: Honoraria **Randy Taplitz, MD**, Karius: Advisor/Consultant|Merck: Advisor/Consultant|SNIPR biome: Advisor/Consultant

